# Revisiting the diagnosis and treatment of Para Kala-azar Dermal Leishmaniasis in the endemic foci of Bangladesh

**DOI:** 10.1371/journal.pone.0280747

**Published:** 2023-01-20

**Authors:** Shomik Maruf, Soumik Kha Sagar, Md. Masud Ur Rashid, Proggananda Nath, Md. Sahidul Islam, Prakash Ghosh, Md. Utba Rashid, Dinesh Mondal, Ahmed Abd El Wahed, Ariful Basher

**Affiliations:** 1 Nutrition and Clinical Services Division, International Centre for Diarrhoeal Disease Research, Dhaka, Bangladesh; 2 National Heart Foundation and Research Institute, Mirpur, Dhaka, Bangladesh; 3 Infectious and Tropical Medicine Department, Mymensingh Medical College and Hospital (MMCH), Mymensingh, Bangladesh; 4 World Health Organization (WHO), Dhaka, Bangladesh; 5 Institute of Animal Hygiene and Veterinary Public Health, University of Leipzig, Leipzig, Germany; 6 Infectious Disease Hospital, Mohakhali, Dhaka, Bangladesh; Iran University of Medical Sciences, ISLAMIC REPUBLIC OF IRAN

## Abstract

Para Kala-azar Dermal Leishmaniasis (Para-KDL) manifests the concomitant presence of Post Kala-azar Dermal Leishmaniasis and Visceral Leishmaniasis and works as a reservoir of infection. The study discusses the cases and their management and aims to address the gaps within existing methods of diagnosis and treatment. This retrospective cross-sectional study discusses 16 Para-KDL cases with one-year follow-up data, treated between 2012–2021 at the Surya Kanta Kala-azar Research Center, Bangladesh. We collected data from hospital records and used STATA 16 to analyze and see the frequency distribution and variable means. We found five patients without any history of kala-azar infection. All the patients were treated with 20 mg/kg Liposomal Amphotericin B in 4 divided doses except one with a history of AmBisome hypersensitivity. One year after treatment, all patients were free from skin lesions, with no hepatosplenomegaly, and observed significant improvement in BMI and hemoglobin levels. The Para-KDL patients are challenging to diagnose, and the relapse and treatment failure leishmania patients might have belonged to this rare group, contributing to their poor prognosis. Therefore, developing an appropriate diagnostic workflow and a new drug regimen is essential to sustain the success of our elimination efforts.

## Introduction

Leishmaniasis, a vector-borne parasitic systemic disease, is caused by more than 20 different species of the genus *Leishmania* and transmitted between vertebrate hosts through the bite of infected female Phlebotomine sandflies. Leishmaniasis is one of those diseases that predominantly affect the socially underprivileged population of rural areas, hence recognized as a Neglected Tropical Disease (NTD) and a major public health problem in tropical, subtropical, temperate, and Mediterranean regions [1]. It usually presents in three different forms: a) Visceral Leishmaniasis (VL), also known as kala-azar (causing fever, hepatosplenomegaly); b) Cutaneous Leishmaniasis (skin lesions, ulcers); c) Mucocutaneous Leishmaniasis (lesions in the mucous membrane). Almost 600 million people living in Leishmaniasis endemic areas are at risk of infection, and about 50000–90000 new cases of VL and more than 1 million new cases of CL are estimated to occur annually worldwide. VL or Kala-azar is the most severe and life-threatening form among all three forms of Leishmaniasis. It occurs most commonly in South Asia (India, Bangladesh, Nepal), East Africa (Sudan, Ethiopia, Kenya), and Brazil. Patients usually present with prolonged high-grade fever, a history of residing or traveling in endemic areas, along with anorexia, weight loss, weakness, hepatosplenomegaly, pancytopenia, etc., and could progress towards fatal consequences if not treated [2].

Prior research has revealed that 10–20% of Kala-azar patients may develop Post Kala-azar Dermal Leishmaniasis (PKDL)—a stigmatizing dermatological disorder. However, it manifests far less mortality compared to VL. PKDL patients present with macular (most common), papular, nodular, or mixed skin rashes. The rashes usually appear in the perioral area and spread gradually toward the neck, chest, trunk, hands, and legs. In the Indian subcontinent, it occurs within 3 years following successful VL treatment, whereas in Africa (mainly Sudan), it appears within 0–6 months among 50–60% of patients, even though PKDL without prior VL history has also been reported [3].

In contrast to Africa, where PKDL cases heal spontaneously without any treatment, PKDL is a major public health concern in South Asia, as it acts as the source of infection and needs treatment. The simultaneous presence of VL and PKDL, regarded as Para Kala-azar Dermal Leishmaniasis, is a common phenomenon in leishmania-endemic African regions [4]. Despite the sporadic reports from India, Para KDL cases are pretty rare in the Indian Sub-continent [5, 6].

Since 2012, sixteen Para-KDL case records have been documented at Surya Kanta Kala-azar Research Centre (SKKRC), Mymensingh, Bangladesh. Patients presented with a variety of features, which made the diagnosis and management difficult, along with the treatment strategy. The detailed clinico-epidemiological profile and treatment are unknown for Para KDL. A study conducted in India reported 24 mutated genes among the Para-KDL strain of *L*. *donovani*, with a possible correlation to developing resistance against sodium stibogluconate (SSG) [7]. Hence, treating these cases with conventional medications used in VL and PKDL treatment may act paradoxically as well as the rate of drug resistance may also increase. Above all, these patients may serve as the super spreader of the disease [8], which in turn hinder the current progression made by the Kala-azar Elimination Program (KEP) toward zero transmission. Therefore, we need new strategies to diagnose such cases and new evidence-based management modalities that would cure patients and mitigate the chance of drug resistance. In this paper, we are reporting a series of cases with their clinic-epidemiological profile and treatment options used.

## Methods

Surya Kanta Kala-azar Research Center (SKKRC) is a tertiary research, referral, and treatment facility of the Government of Bangladesh. Written informed consent are taken from all the admitted patients to use their data for further research in the future by the hospital staff during admission at the SKKRC. We have received all the data anonymously from the hospital (without names, addresses, and other particulars that can be used to identify the patients.) We have also received a letter of permission from the Hospital in-charge to use the data for publication. As this is government data, collected by government staff, using written informed consents the authority and we hope to waive the requirement for an IRB review (which we were granted by other journals (including PLOS NTD). Furthermore, we didn’t receive any funding to carry out this research and we believe this research is of great importance in the post-elimination phase of VL in the Indian Sub-continent. We have extracted data retrospectively from the patient record files collected by hospital staff upon permission from the hospital authority.

SKKRC is situated in Mymensingh District—the most endemic region for leishmania in Bangladesh and designated for the treatment of all kinds of leisnmanisis cases. We conducted a retrospective cohort study through data collection from the hospital records and documented sixteen cases of Para KDL from January 2012 to December 2021. The hospital records included hospitalized patient admission logs and patient information files containing diagnosis and treatment. The hospital staff recorded the data of each patient in the patient information file while they were admitted to the hospital. The diagnosis was made per the Government’s Kala-azar management guideline [16]. New VL cases were diagnosed clinically based on the following criteria—1. Duration of Fever > 2 weeks, 2. Splenomegaly, 3. Residing/ visiting Kala-azar endemic region, and 4. Positive rk39 test. In addition to the aforementioned criteria, the presence of Leishman-Donovan (LD) bodies in microscopy and/or qPCR of splenic aspiration and blood, respectively, were determined for the confirmation of relapse cases. PKDL was diagnosed through the presence of LD bodies in slit skin smear through microscopy and/or qPCR.

Liposomal Amphotericin B (LAmB) with 20 mg/kg body weight in multiple dosages was the treatment of choice for these cases. A patient with drug hypersensitivity to LAmB was treated with Miltefosine at 100 mg/day in two divided doses for 84 days. LAmB is the drug of choice for VL, and while for PKDL, Miltefosine is the preferred drug as per the National Guideline in Bangladesh. All the patients were followed-up for a year after the completion of treatment. Permission was obtained from the hospital authority to use the hospital record files. The retracted data were analyzed to see the frequency distribution and mean of the variables using STATA 16. Additionally, we performed a Chi-squared test to see the differences in parameters at the baseline and end of follow-up.

## Result

### Demographic information of the patients

Analysis of the retrospective data shows a male predominance in the case of Para-KDL, as 13 (81.25%) of the 16 patients were male. Most of the patients (68.75%) were within the 18–45 years of age group, whereas four patients were under 18 years, and only one patient was 60 years old (Table 1).

**Table 1:** Clinical and diagnostic profile and treatment plan of the Para-KDL patients.

Case	Age	Sex	BMI		Fever		Hemoglobin		Spleen	Liver	Fever (month)	Lesion type	Diseases type	Previous history		Previous episode		Last occurrence		Diagnosis		Previous drug		Treatment	Treatment outcome
			Base line	End line	Base line	End line	Base line	End line						PKDL	VL	PKDL	VL	PKDL	VL	PKDL	VL	PKDL	VL		
1	12	M	14.0	18.5	103.0	99.0	10.0	13.0	10	2	5	Mixed	VL_R_ PKDL	Yes	Yes	2	1	2	5	qPCR	M_SA_	LAmB+MF	SSG	LAmB 20 mg	Cured
2	25	F	27.0	27.6	102.0	98.0	13.0	14.0	2	0	2	Macular	VL+PKDL	No	No	-	-	-	-	C_D_	C_D_	-	-	LAmB 20 mg	Cured
3	20	M	18.0	19.5	102.0	98.0	16.0	17.0	2	0	1	Macular	VL+PKDL	No	No	-	-	-	-	C_D_	C_D_	-	-	LAmB 20 mg	Cured
4	38	M	17.0	19.6	101.0	98.0	10.0	12.0	7	2	2	Macular	VL_R_+PKDL	Yes	Yes	1	1	5	6	qPCR	M_SA_	SSG	SSG	LAmB 20 mg	Cured
5	18	M	18.0	19.5	101.0	98.0	7.0	14.0	7	0	3	Macular	VL_R_+PKDL	No	Yes	-	1	-	2	M_SK_	M_SA_	-	SSG	LAmB 20 mg	Cured
6	21	F	17.0	20.4	100.0	99.0	6.0	12.0	4	0	1	Macular	VL_R_+PKDL	No	Yes	-	1	-	5	C_D_	M_SA_	-	MF	LAmB 20 mg	Cured
7	25	M	18.0	19.3	102.0	99.0	10.0	14.0	5	1	1	Macular	VL_R_+PKDL	Yes	Yes	1	1	5	5	qPCR	M_SA_	SSG	SSG	LAmB 20 mg	Cured
8	17	M	15.0	19.6	102.0	98.0	8.0	15.0	5	0	1	Macular	VL_R_+PKDL	No	Yes	-	1	-	3	C_D_	M_SA_	-	SSG	LAmB 20 mg	Cured
9	19	M	17.0	18.7	102.0	98.0	8.0	15.0	6	0	2	Macular	VL_R_+PKDL	No	Yes	-	1	-	3	C_D_	M_SA_	-	LAmB, LAmB	MF 12weeks	Cured
10	60	M	17.0	18.7	103.0	98.0	12.0	12.0	4	1	1	Macular	VL+PKDL	No	No	-	-	-	-	C_D_	C_D_	-	-	LAmB 20 mg	Cured
11	27	M	18.0	19.1	100.0	99.0	10.0	14.0	5	0	2	Mixed	VL_R_+PKDL	Yes	Yes	1	2	1	2	M_SK_	M_SA_	MF	LAmB, LAmB	LAmB 20 mg	Cured
12	10	M	14.0	18.9	101.0	98.0	9.0	13.0	5	0	1	Macular	VL_R_+PKDL	No	Yes	-	1	-	5	qPCR	M_SA_	-	SSG	LAmB 20 mg	Cured
13	24	M	18.0	20.3	101.0	98.0	16.0	17.0	4	0	1	Macular	VL+PKDL	No	No	-	-	-	-	C_D_	C_D_	-	-	LAmB 20 mg	Cured
14	45	M	20.0	21.9	100.0	98.0	12.0	16.0	5	0	2	Macular	VL_R_+PKDL	No	Yes	-	2	-	2	C_D_	M_SA_	-	LAmB	LAmB 20 mg	Cured
15	42	M	15.2		101.0		11.2	14.9	9	0	3	Macular	VL_R_+PKDL	No	Yes	-	2	-	2	qPCR	C_D_	-	SSG, LAmB	LAmB 20 mg	Cured
16	10	F	14.8		102.0		9.2	14.2	25	3	6	Mixed	VL+PKDL	No	No	-	-	-	-	C_D_	C_D_	-	-	LAmB 20 mg	Cured
summary	25.8 ± 13.8	M: n = 13 (81.25%) F: n = 3 (18.75%)	17.4 ± 3.1	20.1 ± 2.3	101.4 ± 1.0	98.3 ± 0.5	10.5 ± 2.8	14.1 ± 1.7	6.6 ± 5.4	0.6 ± 1.0	2.1 ± 1.5	Macular: n = 13 (81.25%) Mixed: n = 3 (18.75%)	VL_R_+PKDL: n = 11 (68.75%) VL+PKDL: n = 5 (31.25%)	No: n = 12 (75%) Yes: n = 4 (25%)	No: n = 5 (31.25%) Yes: n = 11 (68.75%)	1.3 ± 0.5	1.3 ± 0.5	3.3 ± 2.1	3.6 ± 1.6	C_D_: n = 9 (56.25%) M_SK_: n = 2 (12.5%) qPCR: n = 5 (31.25%)	CD: n = 6 (37.5%) M_SA_: n = 10 (62.5%)	SSG: n = 2 (50%), MF: n = 1 (25%), LAmB+MF: n = 1 (25%)	LAmB: n = 6 (42.9%) MF: n = 1 (7.1%) SSG: n = 7 (50%)	LAmB: n = 15 (93.75%) MF: n = 1 (6.25%)	

VL_N_: New VL, VL_R_: VL Relapse, PKDL_N_: New PKDL, PKDL_R_: PKDL Relapse, C_D_: Clinically, M_SK_: Skin Microscopy, M_SA_: Splenic Aspiration Microscopy.

### Information regarding previous episodes of VL and PKDL

Out of the sixteen patients, eleven (68.75%) had a prior history of VL, three had multiple, and the rest had a single episode of VL. None of the patients reported any simultaneous presence of cutaneous lesions during their previous VL episodes. The mean duration between the current Para-KDL and previous VL and PKDL episodes was 3.3 years (2.1) and 3.6 years (1.6), respectively. Six out of eight patients with a single VL episode were treated with Sodium Stibogluconate (SSG), one received Miltefosine, and the remaining patient was treated with Liposomal Amphotericin B (LAmB). Out of three patients with multiple episodes, two got LAmB on both occasions, and the third patient was treated with SSG and LAmB on consecutive episodes. Four Para-KDL patients had a previous history of PKDL, and two received treatment with SSG and one with Miltefosine. The fourth patient had PKDL twice and was treated with Miltefosine and LAmB for the first and second episodes, respectively (Table 1).

### Information regarding Para-KDL

#### Baseline information

All the patients enrolled in this study presented with a history of fever for more than 2 weeks with a mean duration of 2.1 months (1.5), mean temperature of 101.4°F (1.0) (101.4 ± 1.0), and a palpable spleen, averaging 6.6 cm (5.4). Fourteen (87.5%) out of 16 patients were underweight, with a mean BMI of 17.4kg/m^2^ (3.1). Hepatomegaly was present in 5 patients. Cutaneous lesions associated with Para-KDL were predominantly macular on examination of the skin, as 13 patients had hypomelanotic, painless macular lesions. The rest had a mixed picture of macular and nodular lesions (Table 1).

National Guideline for Kala-azar was followed while diagnosing these patients, and both VL and PKDL diagnosis was made separately in each of the patients. Five new cases were diagnosed clinically, and a positive rK39 RDT test. Ten relapse VL cases were confirmed by the presence of LD bodies in splenic aspirate through microscopy. One relapse VL case was provisionally diagnosed clinically and was confirmed of having both visceral and cutaneous infection after qPCR for slit skin smear tested positive. A total of five PKDL cases were diagnosed using skin qPCR, two using positive skin microscopy, and nine PKDL cases were diagnosed clinically.

Fifteen out of the 16 Para-KDL patients were treated with AmBisome 20 mg/kg in 4 equally divided doses. Due to the hypersensitivity reaction, one patient received Cap. Miltefosine 50 mg, twice daily for 12 weeks instead of AmBisome. The patients were discharged after the initial resolution of the VL symptoms and followed up for one year after treatment.

#### End line information

**Fig 1:**
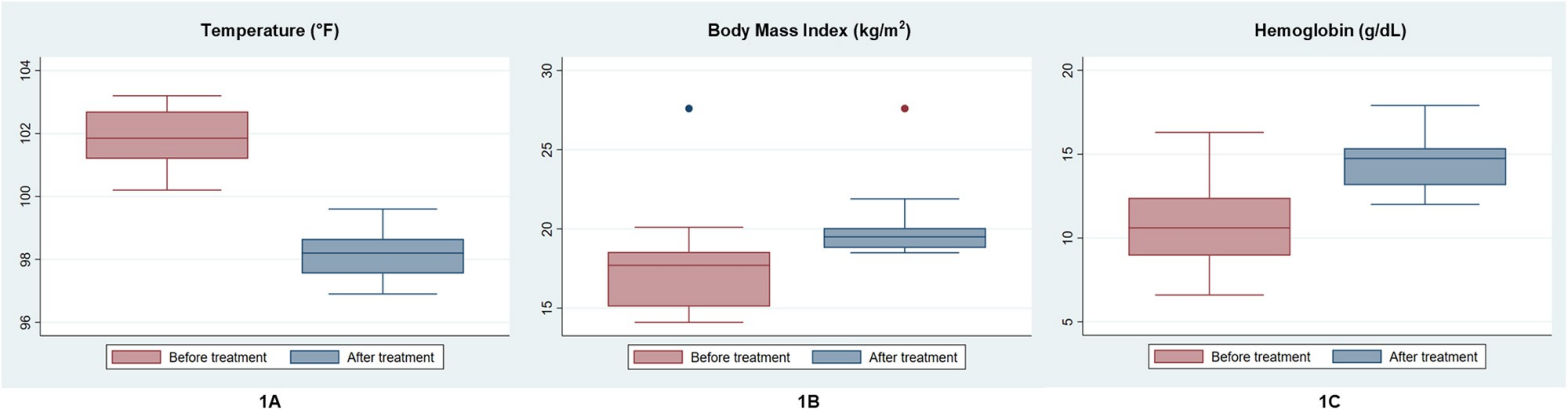
Before and one year after treatment, clinical characteristics of the Para-KDL patients. 1A - mean body temperature (°F), 1B - mean BMI, and 1C - mean Haemoglobin level.

Analysis of the available data after treatment follow-up shows that all the patients had experienced clinical improvement, ensuring complete cure of VL as none had a fever (average temperature 98.3°F, 0.5) (p-value <0.001) (Fig 1A), splenomegaly, or hepatomegaly (Table 1). Also, all of them had substantial weight gain (12.96%) as their mean BMI raised to 19.99 kg/m^2^ from 17.4 kg/m^2^ (Fig 1B). Hematological parameters were also improved significantly (25.5%; p value <0.0001), as the Hemoglobin level rose from 10.5 gm/dl (9.38, 12.31) on the baseline to 14.1 gm/dl (13.66, 15.59) during the follow-up (Fig 1C). There was also clinical improvement in PKDL lesions of all the patients. However, data for the parasitological assessment of PKDL could not be retrieved from the records.

## Discussion

Unlike in East Africa, the simultaneous presence of clinical features for VL and associated skin lesions is a rare event in the Indian subcontinent. However, the establishment of the exact chronology of such presentation is still unknown in both regions. The majority of the patients in this study developed symptoms of VL on a background of PKDL. The five Para-KDL patients without a history of VL might have been asymptomatic careers.

In this study, most of the patients were aged between 18–45 years which is similar to an Indian case series where 6 out of 9 patients were under this age group [9]. The average body temperature and spleen size were, however, higher than that of the Indian case series. Most of the patients presented with macular type PKDL-like lesions—which is the most common type in the Indian subcontinent and resembles other studies as well [9, 10].

In our study, all para-KDL patients had a concoction of clinicopathological pictures, not identical to VL or PKDL alone. These patients presented with a history of fever for weeks to months, constitutional symptoms like weight loss, weakness, hepatosplenomegaly, anemia, and either macular or mixed (macular, papular, and nodular) skin lesions. All the patients were rK39 positive, and none of them were immunocompromised (negative for HIV, HBsAg, anti-HCV, and absence of tuberculosis).

In this study, the majority of the VL cases were diagnosed through the presence of Leishmania donovani (LD) bodies in splenic aspirates under the microscope, which is considered the gold standard with a sensitivity of 85–90% [11]. The sensitivity of LD body demonstration in the skin snip through microscopy is, however, very low in macular lesions (30–40%) [12]. Hence, only two cases were diagnosed through this technique, whereas nine cases were diagnosed clinically. Five cases were diagnosed through qPCR, which is gradually replacing other diagnostic procedures due to its higher sensitivity and specificity [13, 14]. A recently published phase 2 study demonstrated sensitivity of 96.86% and 93.50%, respectively, in diagnosing VL and PKDL cases through the Recombinase Polymerase Amplification (RPA) assay, which is fast, accurate, cost-efficient, comes in a suitcase, and can be established in primary health care facilities [15]. With the emergence of such mobile suitcase laboratories, the PCR test is hoping to revolutionize leishmania diagnosis in the near future.

According to the National Guideline for Kala-azar Management of Bangladesh, single-dose AmBisome of 10 mg/kg in 5% DA over a period of 3–4 hours is recommended for VL treatment. AmBisome in a dose of 21 mg/kg in 5% DA over 2–4 weeks (not more than 10 mg/kg/week) is recommended as an alternative to the first-line treatment in the case of PKDL [16]. VL is the fatal variant of the two, but parasite survival is longer in PKDL than in VL, resulting in the requirement of a higher dose and duration for PKDL treatment. Therefore, all the patients with Para-KDL were treated with AmBisome 20 mg/kg in 4 divided doses except one, who received Cap. Miltefosine for 12 weeks, and both the treatment regimen cover both disease manifestations. The symptoms of VL started to resolve during the hospital stay after the treatment, and patients were clinically well during the after-treatment follow-up. The skin lesions also improved during this period, but exact data on the improvement of skin lesions (Reduction of the number of areas of skin affected, skin microscopy/qPCR results) were unavailable. A case series reported from India treated VL and PKDL separately, using AmBisome to treat VL, followed by Miltefosine to treat PKDL after one month of initial treatment or VL [9].

**Fig 2:**
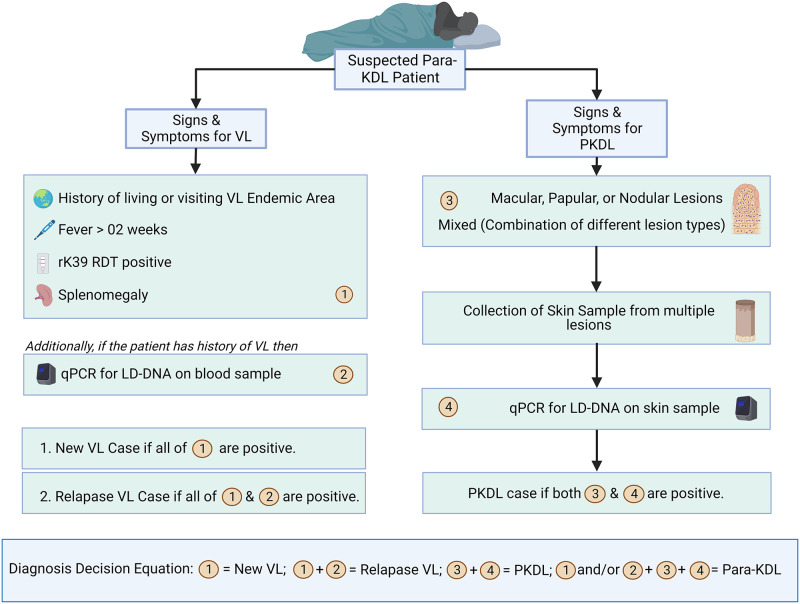
Para-KDL diagnostic workflow.

Despite the recent advancements in the pathogenesis and management of VL and PKDL, the simultaneous presence of VL and PKDL still remain unsolved and warrants urgent attention. The first step to address this problem might be establishing a case definition. Despite the availability of several case definitions with diagnostic criteria in the National Guideline for other types of Leishmaniasis, Para-KDL is still an unaddressed issue at the policy level, which eventually hinders its management strategy. Unlike VL elimination, PKDL elimination is not well addressed in the National Kala-azar Elimination Program (NKEP) in Bangladesh. In addition, there are very few available drugs for the treatment of PKDL. Treating such cases with the concomitant presence of VL and PKDL without any evidence-based management strategy would further increase the chance of drug resistance that could impede our progress toward kala-azar elimination. Our enhanced diagnostic workflow, coupled with PCR for improved diagnostic accuracy of the disease, could be beneficial in such cases (Fig 2).

The patients diagnosed only as either new or relapse VL and PKDL will be treated following the National guideline, which potentially allows the physicians to treat para-KDL patients with either AmBisome or Miltefosine monotherapy. However, AmBisome shows give a better outcome for systemic infection (VL), while Miltefosine has a better skin penetration which helps to resolve PKDL better [4, 17]. Unpublished data from our recently finished clinical trial using a combination of multidose AmBisome (20 mg/kg body weight in 5 divided doses) and oral Miltefosine (allometric dosing BID) capsules for 10 days to treat PKDL cases has proven to be safe and shows promising results *(personal communication*: *Dr*. *Dinesh Mondal)*.

As national guideline was followed for the diagnosis of these patients, detailed clinical and laboratory parameters (especially detailed haemotological profile) were not found in the patient records. New VL and PKDL cases were diagnosed clinically according to the guideline as well; however, parasitological confirmation would have made the diagnosis stronger. Moreover, assessment of PKDL lesion status during the follow up would have also given us a better picture of the cure assessment. Considering the decreased VL patient burden in the Indian Sub-Continent, the Elimination Programs can address these issues and incorporate them into the VL guideline in the future.

## Conclusion

As para-KDL manifests the presence of both systemic and dermal parasitic infection, the combination of these two drugs thus will better serve the purpose of complete parasite clearance. Therefore, we propose a combination of multidose AmBisome and oral Miltefosine capsules in the above-mentioned dosage to treat Para-KDL cases. Nevertheless, ongoing strives to introduce newer drugs for treating Leishmaniasis should be continued to fight the resistance against available drugs and further improvement of case management strategies.
